# Evaluation of commercial ELISA kits’ diagnostic specificity for FAST diseases in wild animals

**DOI:** 10.4102/ojvr.v91i1.2164

**Published:** 2024-06-28

**Authors:** Vesna Milićević, Dimitrije Glišić, Ljubiša Veljović, Jovan Mirčeta, Branislav Kureljušić, Milutin Đorđević, Nikola Vasković

**Affiliations:** 1 Department of Virology, Institute of Veterinary Medicine of Serbia, Belgrade, Serbia; 2 Public Enterprise Vojvodinašume, Novi Sad, Serbia; 3 Department of Pathology, Institute of Veterinary Medicine of Serbia, Belgrade, Serbia; 4 Faculty of Veterinary Medicine, Institute of Veterinary Medicine of Serbia, Belgrade, Serbia; 5 Specialized Veterinary Institute, Kraljevo, Serbia

**Keywords:** diagnostic specificity, ELISA, foot-and-mouth disease, FAST diseases, surveillance, wild ruminants

## Abstract

Wild animals, sharing pathogens with domestic animals, play a crucial role in the epidemiology of infectious diseases. Sampling from wild animals poses significant challenges, yet it is vital for inclusion in disease surveillance and monitoring programmes. Often, mass surveillance involves serological screenings using enzyme-linked immunosorbent assay (ELISA) tests, typically validated only for domestic animals. This study assessed the diagnostic specificity of commercially available ELISA tests on 342 wild ruminant serum samples and 100 from wild boars. We evaluated three tests for foot-and-mouth disease: two for Peste des petits ruminants, two for Rift Valley fever and one for Capripox virus. Diagnostic specificity was calculated using the formula True Negative/(False Positive + True Negative). Cohen’s kappa coefficient measured agreement between tests. Results showed high specificity and agreement across all tests. Specificity for foot-and-mouth disease (FMD) ranged from 93.89% for Prionics to 100% for IDEXX, with IDvet showing 99.6%. The highest agreement was between FMD IDvet and IDEXX at 97.1%. Rift Valley fever (RVF) tests, Ingezim and IDvet, achieved specificities of 100% and 98.83%, respectively. The optimal specificity was attained by retesting single reactors and inactivating the complement.

**Contribution:**

Commercially available ELISA kits are specific for foot-and-mouth disease and similar transboundary animal diseases and can be used for highly specific wild animal testing.

## Introduction

Foot-and-mouth disease and similar transboundary (FAST) animal diseases are viral diseases of ruminants and swine sharing similar clinical signs with foot-and-mouth disease (Gortázar et al. 2022). They include foot-and-mouth disease (FMD), Peste des petits ruminants (PPR), Capripoxviruses (CaPV), Rift Valley fever (RVF), and bovine ephemeral fever (BEF), which are also known as transboundary animal diseases (TADs). Considering their socio-economic impact and the consequences, prevention and surveillance are crucial in non-endemic regions such as Europe. Only in the last decade, European countries, particularly Bulgaria, experienced several outbreaks of FAST diseases: FMD (2011) (Alexandrov et al. 2013), sheep and goat pox (SPGP) (2013-2015) (Tuppurainen et al. 2017), lumpy skin disease (LSD) (2016) (Mercier et al. 2018), and PPR (2018) (Hacıoğlu et al. 2020). Though all were successfully resolved, given the proximity of endemic regions and intensive cross-border movements of humans and animals, the reoccurrence of these diseases cannot be excluded. Rift Valley fever and BEF are vector-borne diseases never reported in Europe. Rift Valley fever is endemic in sub-Saharan Africa, but the outbreaks have been reported in Egypt, Saudi Arabia, and Yemen (Tucker et al. 2020). Bovine ephemeral fever virus has been reported seasonally in Africa, Asia, and Australia (Stokes et al. 2020).

The occurrence of vector-borne diseases in ruminants, such as bluetongue, Schmallenberg, and LSD in Europe, suggests the emergence of arboviruses in the region. The concern is growing due to the undetermined incursion routes, raising the possibility of introducing and spreading Rift Valley fever (RVF) and bovine ephemeral fever virus (BEFV) in Europe. Besides domestic animals, wild animals are also susceptible to FAST diseases (Gortázar, Ruiz-Fons & Höfle 2016). Recognising their potential role as sentinels and reservoirs in disease epidemiology, the surveillance of FAST diseases in wildlife should be an integral aspect of the overall strategy for surveillance and disease control (Gortázar et al. 2022). Diagnosing FAST diseases in non-endemic regions typically relies on detecting and identifying agents, predominantly using molecular methods.

However, enzyme-linked immunosorbent assay (ELISA) tests for antibody detection are commonly used to establish disease-free status. The commercial ELISA kits may be optimised to be of high diagnostic sensitivity (DSe) with associated lower diagnostic specificity (DSp) for screening purposes or with high diagnostic specificity but lower sensitivity for the confirmatory assays. The estimated DSe and DSp are primary inputs for calculating sample size for freedom testing using imperfect tests and predictive values of positive and negative test results (Jia et al. 2020). For accurate DSe and DSp estimation, reference animals with known disease history are required. Negative reference animals are typically sourced from regions or countries free from the disease. However, animals from non-infected or disease-free zones in endemic areas can also be utilised. Usually, commercial kits are validated using domestic animal samples, considering the availability and ease of obtaining them. However, in the context of wild animals’ role in the epidemiology of FAST diseases and the need to test them, accurate information on the DSe and DSp of ELISA kits in wild animals is also required. Because FMD and SPGP are absent, and PPR and RVF have never been documented in Serbia according to the World Animal Health Information System (WAHIS), samples collected from wild animals can serve as a negative reference to validate the data regarding the diagnostic specificity of commercially available ELISA kits. Thus, the main aim of this study was to provide scientific information on the diagnostic specificity of commercially available ELISA kits and their utility in testing wild animals.

## Material and methods

### Samples collection

A systematic sampling effort was undertaken to gather specimens from various wild animals, including red deer (*Cervus elaphus*), fallow deer (*Dama dama*), mouflon (*Ovis aries musimon*), and roe deer (*Capreolus capreolus*). This initiative was carried out during the 2022–2023 hunting season. Though most species’ hunting season extends from summer to spring, the season’s peak is during winter, particularly in December and January. The duration of the hunting season, defined in the Regulation on amendments to the Rulebook on the declaration of wild game species protected by wildlife (Official Gazette number 92 of 09/22/2021), is given in Table 1.

**TABLE 1:** Duration of hunting season for selected species and categories.

Species (common names)	Species (Latin name)	Category	Duration of hunting	Months																							
				January		February		March		April		May		June		July		August		September		October		November		December	
				1–15	16–31	1–15	16-28(29)	1–15	16–31	1–15	16–30	1–15	16–31	1–15	16–30	1–15	16–31	1–15	16–31	1–15	16–30	1–15	16–31	1–15	16–30	1–15	16–31
Wild boar	Sus scrofa	Boar	15 April to 28 February	√	√	√	√	X	X	X	√	√	√	√	√	√	√	√	√	√	√	√	√	√	√	√	√
		Sow	01 July to 15 January	√	X	X	X	X	X	X	X	X	X	X	X	√	√	√	√	√	√	√	√	√	√	√	√
		Piglet	15 April to 28 February	√	√	√	√	X	X	X	√	√	√	√	√	√	√	√	√	√	√	√	√	√	√	√	√
Mouflon	Ovis aries musimon	Ram	01 January to 31 December	√	√	√	√	√	√	√	√	√	√	√	√	√	√	√	√	√	√	√	√	√	√	√	√
		Ewe and lamb	01 October to 31 January	√	√	X	X	X	X	X	X	X	X	X	X	X	X	X	X	X	X	√	√	√	√	√	√
Red deer	Cervus elaphus	Stag	01 August to 15 February	√	√	√	X	X	X	X	X	X	X	X	X	X	X	√	√	√	√	√	√	√	√	√	√
		Hind and calf	01 August to 15 February	√	√	√	X	X	X	X	X	X	X	X	X	X	X	√	√	√	√	√	√	√	√	√	√
Fallow deer	Dama dama	Buck	01 September to 15 February	√	√	√	X	X	X	X	X	X	X	X	X	X	X	X	X	√	√	√	√	√	√	√	√
		Doe and fawn	01 September to 15 February	√	√	√	X	X	X	X	X	X	X	X	X	X	X	X	X	√	√	√	√	√	√	√	√
Roe deer	Capreolus capreolus	Buck	15 April to 30 September	X	X	X	X	X	X	X	√	√	√	√	√	√	√	√	√	√	√	X	X	X	X	X	X
		Doe and kid	01 September to 31 January	√	√	X	X	X	X	X	X	X	X	X	X	X	X	X	X	√	√	√	√	√	√	√	√

*Source*: Srbija šume, 2024, *Kalendar lova*, viewed n.d., from https://srbijasume.rs/en/lovstvo/kalendar-lova/

√, open hunting season; X, closed hunting season.

Blood samples from wild ruminants were collected in collaboration with the public enterprise ‘Vojvodinašume’ and local hunters’ associations, all conducted in compliance with the relevant legal regulations. Importantly, ethical or animal welfare approval was not necessary because the samples were obtained *post mortem* by the hunters. The collection process involved drawing blood from the heart using a syringe and needle. Hunters were provided with guidance on proper sampling techniques and a submission form, which included essential information such as species, sex, age, and geographic coordinates. Each sample was carefully labelled and accompanied by a submission form containing identifying details on geolocation, species, age, and sex. The hunters determined the animals’ age based on the number of permanent teeth and the extent of dental wear. In the case of wild boar (Sus scrofa), the blood samples were sourced from an ongoing state surveillance programme for African and classical swine fever. The age of the harvested wild boars was determined by assessing their dentition characteristics, adhering to the guidelines provided by SC (EDA SCHEDA Ecological Associates, Inc.) as described below: boars aged 0–6 months have no permanent molars; those aged 6–18 months have one permanent molar; individuals aged 1.5–2.5 years possess two permanent molars; and boars older than 2.5 years exhibit three permanent molars. The determination of the required sample size was calculated using Survey Toolbox software, aiming for a 5% prevalence, 2% error and 99% confidence. As a result, the plan involved collecting and testing 325 samples from wild ruminants and 100 samples from wild boar, recognising the varying susceptibility of different species to different FAST diseases. Table 2 presents the official count of designated wild animals in Serbia, alongside the tally of harvested ones, recorded using hunting bags, which serve as essential instruments for data collection and wildlife population monitoring.

**TABLE 2:** Estimated number of selected wild species and the hunting bag.

Species	Number of animals	Hunting bag
Red deer	7114	984
Fallow deer	1333	188
Roe deer	143 076	11 454
Mouflon	621	43
Wild boar	25 724	15 228

*Source:* Statistical Office of Serbia, 2021, *Wildlife population*, viewed n.d., from https://data.stat.gov.rs/Home/Result/13040601?languageCode=sr-Latn

### Enzyme-linked immunosorbent assay testing

After the reception in the laboratory, the blood samples were centrifuged 10 min at 1500 g, and sera were decanted and stored at –80 °C until testing. For FMD, three commercial ELISA kits were used: FMD Multispecies Antibody Test Kit, IDEXX – FMD Idexx; ID Screen^®^ FMD non structural protein (NSP) Competition, IDvet – FMD IDvet; PrioCHECK™ foot-and-mouth disease virus (FMDV) NS Antibody ELISA Kit, Prionics – FMD Prionics following the manufacturers’ instructions. The evaluated commercial kits for PPR (ID Screen^®^ PPR Competition, IDvet – PPR IDvet, and INgezim PPR Compac, Ingenasa – PPR Ingezim) were run following the manufacturers’ instructions. For RVF, ID Screen^®^ Rift Valley Fever Competition Multi-species, IDvet – RVF IDvet, and INgezim Fièvre de la vallée du Rift (FVR) Compac, Ingenasa – RVF Ingezim, kits were used following the manufacturer’s instructions. For SPGP, commercial ELISA kit ID Screen^®^ Capripox Double Antigen Multi-species, ID vet – CaPV IDvet was used following the manufacturer’s instructions. After the initial testing, the positive samples were retested. The positive samples were inactivated at 56 °C for 30 min to mitigate non-specific reactions, a common issue attributed to the low quality inherent in wildlife samples. This quality variability often arises from differing storage durations and the compromised condition of samples obtained from shot animals.

Diagnostic specificity was estimated using the formula True Negative/(False Positive + True Negative), considering all samples to be true negative. The obtained results were analyzed using FreeCalc version 2.0 software. Cohen’s kappa coefficient was used to estimate the degree of agreement between the two tests.

### Ethical considerations

Ethical clearance waiver to conduct this study was obtained from the Scientific Institute of Veterinary Medicine of Serbia Institutional Review Board.

## Results

**FIGURE 1:**
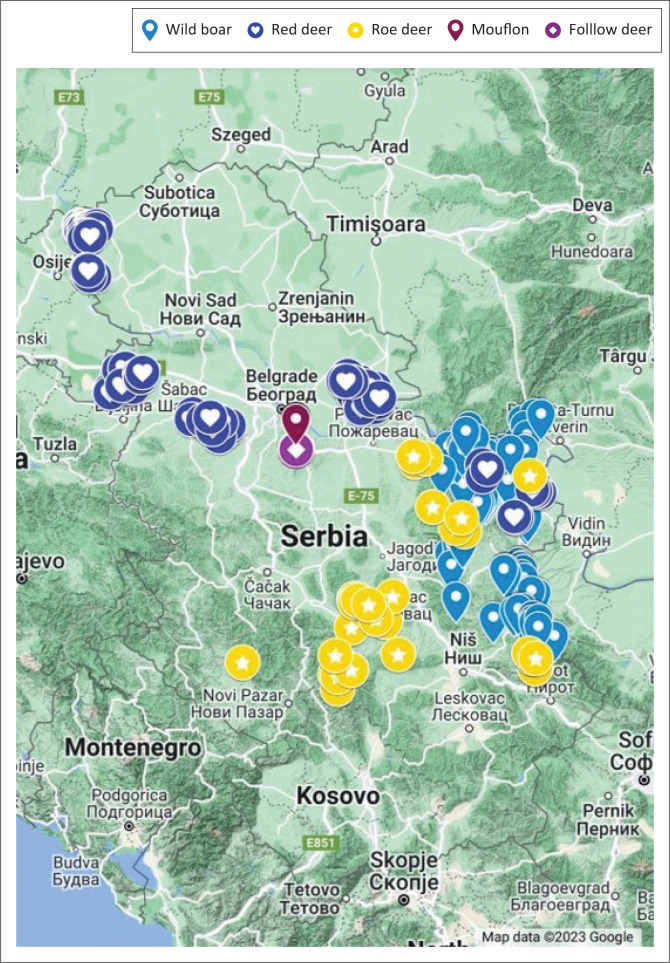
Locations from which wild ruminants and wild boars were sampled.

Across Serbia’s geographic expanse (Figure 1), a comprehensive collection of 100 samples from wild boars (Table 3) and 342 samples from wild ruminants (Table 4) was systematically acquired.

**TABLE 3:** Number, sex, and age of collected wild boars.

Species	Number of collected samples	Sex				Age (in years)							
		Male	%	Female	%	0–0.5	%	0.5–1.5	%	1.5–2.5	%	> 2.5	%
Wild boar	100	55	55	45	45	2	2	61	61	13	13	24	24

**TABLE 4:** Number, sex and age of collected wild ruminants.

Species	Number of collected samples	%	Sex						Age (in years)							
			Male	%	Female	%	Unknown	%	Juvenile, up to 1 year	%	Subadult, 1–2 years	%	Adult, above 2 years	%	Unknown	%
Red deer	252	73.7	138	54.8	93	36.9	21	8.3	12	4.8	30	11.9	190	75.4	20	7.9
Fallow deer	5	1.5	2	40.0	3	60.0	0	0	0	0.0	1	20.0	4	80.0	0	0.0
Roe deer	77	22.5	51	66.2	26	33.8	0	0	10	13.0	9	11.7	56	72.7	2	2.6
Mouflon	8	2.3	2	25.0	6	75.0	0	0	1	12.5	2	25.0	5	62.5	0	0.0

**Total**	**342**	**-**	**193**	**56.5**	**128**	**37.4**	**21**	**6.1**	**23**	**6.7**	**42**	**12.3**	**255**	**74.6**	**22**	**6.4**

Among wild ruminants, most samples originated from red deer (73.7%), followed by roe deer (22.5%), mouflons (2.3%) and fallow deer (1.5%). Among the total sample population, wild boars accounted for 22.6%. According to the age categories, most wild ruminants were sub-adults and adults (86.9%). The animals between 0.5 years and 1.5 years were most sampled (61%) from wild boar.

Foot-and-Mouth Disease Multispecies Antibody Test Kit, IDEXX, INgezim RVF Compac, Ingenasa, INgezim PPR Compac, Ingenasa and PPR, ID Screen^®^ PPR Competition showed the highest DSp considering results after the first test (Table 5).

**TABLE 5:** Estimation of commercial enzyme-linked immunosorbent assay kits diagnostic specificity for wild animals testing.

Commercial ELISA test	Test phase	Results (no.)			DSp (%)
		Positive	Negative	Doubtful	
PrioCHECK™ FMDV NS Antibody ELISA Kit, Prionics	1st test	27	415	0	93.89
	2nd test	9	433	0	97.96
	After inactivation 56 °C, 30 min	3	439	0	99.32
ID Screen® FMD NSP Competition, IDvet	1st test	10	332	0	97.08
	2nd test	4	338	0	98.83
	After inactivation 56 °C, 30 min	2	340	0	99.42
Foot-and-Mouth Disease Multispecies Antibody Test Kit, IDEXX	1st test	0	342	0	100.00
INgezim FVR Compac, Ingenasa	1st test	0	342	0	100.00
ID Screen® Rift Valley Fever Competition Multi-species, IDvet	1st test	4	338	0	98.83
	2nd test	4	338	0	98.83
	After inactivation 56 °C, 30 min	1	341	0	99.71
ID Screen® PPR Competition, IDvet	1st test	0	340	2	99.42
	2nd test	0	342	0	100.00
	After inactivation 56 °C, 30 min	0	342	0	100.00
INgezim PPR Compac, Ingenasa	1st test	0	342	0	100
ID Screen® Capripox Double Antigen Multi-species, ID vet	1st test	5	337	0	98.54
	2nd test	1	341	0	99.71
	After inactivation 56 °C, 30 min	0	342	0	100.00

ELISA, enzyme-linked immunosorbent assay; FMD, foot-and-mouth disease; PPR, peste des petits ruminants; NS, non structural; NSP, non structural protein; DSp, diagnostic specificity; FMDV, foot-and-mouth disease virus.

The difference in results obtained according to species was observed for FMD Prionics and FMD IDvet, which were 100% specific for wild boar but 92.11% and 97.08% for wild ruminants’ samples.

After the initial testing, the lowest DSp was calculated for FMD Prionics (93.89%). However, after the retest, DSp was increased to 97.96%, whereas by testing inactivated serum samples, the DSp reached 99.32%. Foot-and-mouth disease IDvet showed a DSp of 99.6% after the serum inactivation, while the DSp of FMD IDEXX was 100% after the first test. The highest agreement of results was estimated for FMD IDvet and FMD IDEXX (97.1%). Good agreement was also established between FMD Idexx and FMD Prionics (92.1%) and FMD IDvet and FMD Prionics (92.7%, Cohen’s k = 0.29).

After the first test, the DSp of RVF Ingezim was 100%. Rift Valley fever IDvet DSp was 98.83% after the first test and retest. However, DSp after serum inactivation was 99.71%. Thus, the agreement of the results after the initial test was 98.8%.

Compared to 100% DSp of PPR Ingezim, DSp of PPR IDvet was the highest after the serum inactivation, although high after the test and retest (99.42%). The agreement between tests was estimated at 99.4%. Like other tests, CaPV IDvet had the lowest DSp after the first test (98.54%), which increased to 100% after the serum inactivation.

Based on the analysis of single reactors for FMD IDvet, FMD Prionics, and RVF IDvet, utilising calculated diagnostic specificity, a prevalence of 1%, and a specificity of 99%, the findings indicate that the population is confidently determined to be free from the disease at a 100% confidence level, as determined by the FreeCalc software.

## Discussion

This study aimed to estimate the diagnostic specificity of commercially available ELISA kits for FAST diseases in wild animals. European countries are at constant risk of introducing FMD and other FAST diseases due to their occurrence in neighbouring countries. Therefore, the focus lies on monitoring, disease control, and gathering epidemiological data from nations facing outbreaks. This facilitates the acquisition of a clearer and more precise assessment of the associated risks. European countries maintain a high level of preparedness to handle disease incursions, even though the primary goal is to reduce the risk initially. Considering preparedness, knowing the performances of diagnostic methods is one of the crucial factors to be estimated in inter-epidemic period because most laboratories have little or no experience in routine diagnostics of FAST disease (Pérez-Ramírez et al. 2020).

Given that wild animals are also susceptible and can contribute to the maintenance of the disease, the diagnostic tests should also be validated for wildlife population and evaluated for the possibility of use in screening purposes. While specific FAST pathogens may not visibly affect the health of wild animals, studying them in this context is essential. Because species-specific ELISA tests for wild animals are usually lacking, the investigations are limited to competitive and blocking tests intended for multiple domestic species and are usually not validated for wildlife. Thus, interpretation of results should be performed cautiously (Garnier et al. 2017). In addition, when including wild animals in screening programmes, it should be noted that repeated sampling is not possible, and the quality of the sample can be subpar due to haemolysis or repeated freeze-thaw cycles (Boadella & Gortázar 2011).

This study’s main limitation was the lack of gold-standard results, which necessitated classifying samples as negative a priori based on the absence of the diseases in Serbia. However, considering the diagnostic specificity of tests and epidemiological calculation based on predefined 1% prevalence, the results supported the assumption of Serbian-free status of selected diseases in wildlife populations despite single reactors detected. Furthermore, all tested ELISA kits showed diagnostic specificity, which meets the standards of a minimum of 90% (Jacobson 1998). In this study, it was shown that the specificity of a test could be improved by retesting the same sample, but the maximal DSp was obtained after complement inactivation at 56 °C for 30 min. Because this pattern was evident for all tested ELISA kits, the complement inactivation could be performed before the initial screening test as it does not alter antibody titre or the ELISA results but saves time and preserves samples from unnecessary manipulation, as recommended for classical swine fever routine serology tests (Meyer, Petrov & Becher 2019).

In this investigation, three commercially available tests for FMD were assessed. They are intended for anti-non-structural protein (NSP) antibody detection but use different methodologies. Foot-and-mouth disease IDEXX kit is an indirect ELISA test, FMD Prionics is a blocking ELISA, FMD IDvet is competitive, whereas only FMD Prionics could be used for swine testing. Interestingly, all kits showed good performance and high agreement. FMD Prionics was 100% specific for wild boar but 92.11% for wild ruminants, with DSp increasing to 99.12% after the retest and complement inactivation. Absolute specificity for FMD Priocheck was also reported by Chen et al. (2011) for domestic swine samples. Similar results to current study obtained for wild ruminants were reported for cattle: specificity of FMD Prionics was 99.39%, and FMD IDvet in the range of 99.29% to 99.50% (Tewari et al. 2021). Improvement of FMD Prionics specificity from 98.1 to 99.2% with retesting was also reported by Tewari et al. (2021). Thus, the obtained results meet the 97% – 98% requirement for detecting even carriers (Brocchi et al. 2006). Considering the presented results, many false positive reactions could be expected in massive surveillances requiring confirmation of each positive test. NSP ELISA tests are the primary tool in maintaining and re-gaining free status. Virus Neutralisation Test (VNT) or Liquid-phase Blocking ELISA (LPBE) could be used for confirmation. However, the comparison of the results can be difficult because of the difference in time when antibodies became detectable. However, Chitray et al. (2018) suggested that a single NSP test positive can be assumed as a false positive due to the test imperfection. Although wild European species of ruminants and wild boar are susceptible to FMDV, no scientific proof exists of their role in Europe’s most recent FMD outbreaks (Gortázar et al. 2022). The only evidence of FMD in wildlife was the seroprevalence of 7% in wild boar and 4% in roe deer in Bulgaria after the FMD outbreak in 2011, with no virus detection (Alexandrov et al. 2013).

The role of wild animals in PPR epidemiology, considered one of the most threatening transboundary infectious diseases, is assumed minor (Banyard & Parida 2016). However, one of the main recognised research gaps with PPR is the need for validated and standardised diagnostic tools for wildlife (Fine et al. 2020). Peste des petits ruminants IDvet and PPR Ingezim were evaluated for PPR serology diagnostics in wild ruminants. The PPR Ingezim utilises a blocking ELISA methodology, whereas PPR IDvet is a competition ELISA that detects anti-PPRV nucleoprotein antibodies. In the current study, both tests showed high agreement and very good specificity. PPR IDvet kit showed the same pattern of increasing specificity after inactivating and retesting the samples when specificity reached 100%. Absolute specificity for PPR Ingezim was observed almost after the initial test.

However, Lelisa et al. (2022) reported significantly lower specificity (76.36%) than the gold standard. Higher specificity and agreement were observed for goat and sheep samples than cattle samples, whereas the kit was considered unsuitable for camels. For the PPR Ingezim ELISA kit there is very scarce data on its performance. Although RVF is limited to sub-Saharan Africa and the Arabian Peninsula, with climate change enabling vector expansion, the risk of RVF’s introduction into the EU is very low (Nielsen et al. 2020). However, after the reports on seropositive animals in Turkey, Tunisia and Libya, the authorities are recommended to revise surveillance and improve capacities (Nielsen et al. 2020). In this context, two ELISA kits were evaluated: competitive RVF IDvet and blocking RVF Ingezim. Both kits showed good diagnostic specificity and 98.8% agreement in results when used for wild ruminants. Almost the same DSp of 98.1% for RVF IDvet was also reported by De Bronsvoort et al. (2019). Other, recently developed, ELISA kits relying on rNp (Pawęska et al. 2021) also exhibited commendable diagnostic specificity, depending on the disease’s presence. In regions with endemicity, specificity reached 98.6% for cattle and 99.5% for sheep, while in free countries, specificity rates were estimated at 97.7% for sheep and 98.1% for goats (Pawęska et al. 2021). Comparing the kits’ performances based on interlaboratory comparison trials, RVF Ingezim showed good specificity but lower sensitivity, whereas RVF IDvet was superior in diagnostic sensitivity (Pedarrieu et al. 2021).

Because it is only commercially available, one kit was evaluated for Capripox virus antibody detection in wild animals. This double antigen recognition kit was developed after the LSD outbreak in the Balkans in 2015 (Mercier et al. 2018). The estimated DSp was 98.54%, increasing to 99.71% after the retest and 100% after the complement inactivation. Its performance, specificity and sensitivity were reported previously for cattle and mass screening (Milovanović et al. 2019a). Sharing more than 90% homology and being based on recombinant antigen, CaPV IDvet kit can be used to diagnose Lumpy skin disease (LSD) and goat and sheep pox virus infections. At the same time, it is not cross-reacting with Parapox viruses that are widely present in sheep in Serbia (Milovanović et al. 2019b). Recently developed indirect and competitive ELISA for LSD showed a specificity of 99.5% and 100%, regardless of the species tested (Baselli et al. 2023).

Although there is no perfect test, the accuracy of the results interpretation must be indisputable. Depending on the sources, this can be obtained using confirmatory tests (Tewari et al. 2021) or combining tests of different characteristics and methodologies.

## Conclusion

This work contributes significantly to the scientific knowledge of diagnostic specificity of commercially available ELISA kits for selected FAST diseases in wildlife. These results should complement the contingency plans and prescribed diagnostic procedures regarding wildlife inclusion in surveillance and results assessment. In addition, insight into the epizootiology of FAST disease in Serbia is provided by confirming the absence of selected diseases in the area tested.
